# Preoperative intensity-modulated radiotherapy with a simultaneous integrated boost combined with Capecitabine in locally advanced rectal cancer: short-term results of a multicentric study

**DOI:** 10.1186/s13014-017-0870-4

**Published:** 2017-08-22

**Authors:** Marco Lupattelli, Fabio Matrone, Maria Antonietta Gambacorta, Mattia Osti, Gabriella Macchia, Elisa Palazzari, Luca Nicosia, Federico Navarria, Giuditta Chiloiro, Vincenzo Valentini, Cynthia Aristei, Antonino De Paoli

**Affiliations:** 1 Radiation Oncology Dept., Santa Maria della Misericordia Hospital, Piazzale Menghini, 1 - 06129 Perugia, Italy; 2 Radiation Oncology Dept., Santa Maria della Misericordia Hospital - University, Perugia, Italy; 3 Radiation Oncology Dept., CRO - National Cancer Institute, Aviano, Italy; 4 Radiation Oncology Dept., Catholic University of Sacred Heart, Rome, Italy; 5 Radiation Oncology Dept., Sant’ Andrea Hospital - Sapienza University, Rome, Italy; 6 Radiation Oncology Dept., Catholic University of Sacred Heart, Campobasso, Italy

**Keywords:** Imrt-sib, Capecitabine, Pre-operative chemo-radiotherapy, Rectal cancer

## Abstract

**Background:**

Preoperative radiotherapy (RT) in combination with fluoropyrimidine-based chemotherapy (CT) is the standard of care in patients with locally advanced, T3-T4 N0–2, rectal cancer (LARC). Given the correlation between RT dose-tumor response and the prognostic role of the tumor regression grade (TRG), treatment intensification represents an area of active investigation. The aim of the study was to analyze the role of RT dose-intensification in the preoperative treatment of LARC in terms of feasibility, efficacy and toxicity.

**Methods:**

We retrospectively analyzed patients with LARC treated with intensity-modulated radiotherapy (IMRT) and simultaneous integrated boost (SIB) at five Italian radiation oncology centers. Concurrent Capecitabine was administered. Treatment response was evaluated in terms of disease down-staging and TRG. Acute toxicity was evaluated according to the CTC-AE 4.0 scale.

**Results:**

A total of 76 patients were identified for this analysis. A dose of 45 Gy was prescribed to the entire mesorectum and pelvic lymph nodes with a median SIB dose of 54 Gy (range 52.5–57.5) to the tumor and corresponding mesorectum. Overall, 74/76 (97.4%) patients completed the planned RT, whereas 64/76 (84.2%) patients completed the prescribed CT. Eight (10.5%) patients developed grade 3–4 acute toxicity. Overall, 72/76 patients underwent surgery. The tumor and nodal down-staging was documented in 51 (70.8%) and 43 (67%) patients, respectively. Twenty (27.8%) patients obtained a pathologic complete response. Surgical morbidity was reported in 13/72 patients (18.1%).

**Conclusions:**

Although retrospective in design, this study indicates that IMRT-SIB with a dose range of 52.5–57.5 Gy (median 54 Gy) and concomitant Capecitabine appears feasible, well tolerated and effective in terms of disease down-staging and pathological complete response. Long-term toxicity and the impact on disease control and patient survival will be evaluated with a longer follow-up time.

**Trial registration:**

NA

## Introduction

Preoperative radiotherapy (RT) alone or in combination with chemotherapy (CT), is the standard treatment in patients with extra-peritoneal locally advanced rectal cancer (LARC). In patients with unresectable or resectable disease, where “downsizing” and “downstaging” are required (cT3–4 mesorectal fascia [MRF] +/N0–2 or cT3 MRF −/+ N0 of the lower rectum) combination therapy is recommended [1]. Although this multimodality approach leads to an improvement in local control as well as in a significantly higher rates of complete pathological response (pCR), ranging from 13% to 20%, 25–30% of these patients will experience metastatic disease [1]. The evidence that pCR after preoperative chemoradiotherapy (CT-RT) represents an independent favourable prognostic factor for local control, overall survival (OS) and disease free survival (DFS) [2], led to evaluate the role of CT-RT intensification. Treatment intensification with the concurrent CT was evaluated in five randomized trials adding Oxaliplatin to either 5FU or Capecitabine [3–7]. Only one of these studies demonstrated improved outcomes, whereas all studies reported a significantly increased toxicity [4]. Conversely, the evidence of a dose-response relationship in terms of local control and tumor regression grade for RT doses > 50–60 Gy [8, 9] as well as radiobiological data [10] provide a strong rationale for intensified RT with the delivery of higher doses in a shorter time. The favourable results reported by a number of phase I-II studies, evaluating the role of RT intensification with several accelerated fractionation schedules combined with 5FU-based CT in LARC, support these observations [11, 12]. More recently, a phase III randomized trial (INTERACT Trial), which compared radiation dose intensification with 3D conformal RT (3D–CRT) using a concomitant boost technique and Capecitabine versus standard RT and CT intensification by adding Oxaliplatin to Capecitabine, showed no difference in terms of pCR, local control and survival. Conversely, the study reported a statistically significant difference in terms of compliance and acute toxicity in favour of the intensified RT arm [13].

In order to evaluate the role of RT intensification, a number of phase II studies with preoperative intensity-modulated RT (IMRT) have documented the benefit in terms of feasibility and toxicity of simultaneous integrated boost (SIB) intensification in combination with fluoropyrimidine-based CT in patients with LARC. Favourable short and long-term results were reported [14–16]. Accordingly, in this pooled analysis we aimed to retrospectively analyze the data of LARC patients treated with intensified preoperative RT (IMRT-SIB) and Capecitabine in terms of feasibility, toxicity and efficacy.

## Materials and methods

### Inclusion criteria

Patients from five Italian radiation oncology centers with a histological diagnosis of extra-peritoneal LARC (stage II-III), were included in the study.

Information regarding clinical history, physical examination, digital rectal examination, CEA determination, blood profile, and staging exams including colonscopy with biopsy, chest and abdomen CT-scan, endoscopic ultrasound and pelvic MRI were gathered. In particular the MRF involvement was assessed according to the MERCURY study group criteria [17].

IMRT was delivered to the entire mesorectum and obturator, presacral, internal iliac lymph nodes (plus external iliac lymph nodes in the cT4 patients) (CTV1) with SIB to the tumour and corresponding mesorectum plus an MRI-based cranio-caudal extension of 1–2 cm (CTV2). Concomitant Capecitabine was administered. Organs at risk constraints and prescribed dose as well as surgical and pathological details after operation, including the standardized five-points tumour regression grade (TRG) according to Mandard et al. [18] had to be available. A pCR was defined as no visible microscopic disease in the primary tumour and lymph-nodes. All patients enrolled in the analysis had to report at least one month of follow-up after surgery. The study was approved by the Internal Review Boards of the participating centers.

### Objectives of the study

Our purpose was to determine the feasibility, efficacy and toxicity of RT intensification with IMRT-SIB and concomitant Capecitabine. The feasibility was assessed on the basis of compliance with the proposed treatment, by evaluating RT and concomitant CT separately. Efficacy was defined by tumour and nodal down-staging and TRG according to the Mandard score [18]. Acute toxicity was evaluated according to the CTC-AE 4.0 scale. Post-operative morbidity was evaluated after 30 days from surgery.

### Statistical analysis

The Chi-square test was used to compare data of tumor response (downstaging) between different IMRT-SIB doses given (52.5Gy vs 54Gy vs 55Gy vs 57.5Gy). Odds ratio (OR) and 95% confidence interval (CI) for TRG1(pCR) and downstaging were calculated using logistic regression model, adjusting for sex, age, distance of the lower tumor pole from external anal sphyncther (EAS), clinical tumor stage (cT), clinical nodal stage (cN), MRF involvement and RT dose intensity levels, when appropriate.

## Results

### Patient population

From October 2013 to May 2016, 76 patients were included in the analysis. The majority of patients (97.4%) were in good general condition (ECOG 0–1), without significant co-morbidities. In 63 out of 76 patients (83%) tumour localization was in the lower rectum (≤ 6 cm from EAS), and in 71% the tumor was defined as a tethered mass at digital rectal examination. Most cases were stage IIIB (64.5%) and MRF involvement was documented in 45% of patients. Patient, tumor characteristics and RT doses are reported in Table 1.

**Table 1:** Patient and tumor characteristics

	n *(%)*
Sex	
Male	50 (*65.8)*
Female	*26 (34.2)*
Median age *(range)*	64 yrs. *(29–84)*
Performance status	
*ECOG 0–1*	74 *(97.4)*
*ECOG 2*	2 *(2.6)*
Median tumor size *(range)*	50 mm *(25–120)*
Median tumor distance-EAS *(range)*	60 mm *(10–120)*
Tumor Stage	
cT2	5 *(6.5)*
cT3	63 *(82.9)*
cT4	8 *(10.6)*
cN	
cN0	12 *(15.8)*
cN1	39 *(51.3)*
cN2	25 *(32.9)*
Stage	
IIA	12 *(15.8)*
IIIA	3 *(3.9)*
IIIB	49 *(64.5)*
IIIC	12 *(15.8)*
MRF involvement	
Yes	34 *(44.7)*
No	42 *(55.3)*
IMRT-SIB dose (dose per fraction)	
52.5 Gy (2.1Gy)	16 *(21)*
54 Gy (2.16Gy)	24 *(31.5)*
55 Gy (2.2Gy)	34 *(45)*
57.5 Gy (2.5Gy)	2 *(2.5)*

*MRF* Mesorectal fascia

### Treatment compliance and acute toxicity

All patients received a prescription of 45 Gy in 25 fractions to the CTV1. The dose to CTV-SIB (CTV2) ranged from 52.5Gy, 2.1Gy/fraction (16 patients), 54Gy, 2.16Gy/fraction (24 patients), 55Gy, 2.2Gy/fraction (34 patients) to 57.5 Gy, 2.3Gy/fraction (2 patients) corresponding to an equivalent dose at 2 Gy/fraction (EQD2) of 53.24, 55.22Gy, 56.55Gy, to 59.94Gy respectively, (considering α/β = 5.06 Gy for rectal tumor [16]). The median dose was 54 Gy (EQD2: 55.22 Gy). The CTV-SIB dose was a choice of the cancer center; all patients were treated equally at their respective centers. Preoperative IMRT-SIB was given with “step and shoot”, “sliding window”, VMAT and Tomotherapy techniques in 24%, 28%, 44% and 4% of patients, respectively. Overall, 74 out of 76 (97.4%) patients completed IMRT-SIB as planned. Capecitabine at a dose of 1650 mg/m2/daily for the entire course of the RT was administered in 64 of 76 (84.2%) patients. The remaining 12 patients interrupted CT due to haematological (4), gastrointestinal toxicity (3) or other causes (5). Therefore compliance to IMRT-SIB and concurrent Capecitabine was 97.4% and 84.2%, respectively.

Major G3–4 toxicity was reported in 8 patients (10.5%). Gastrointestinal and haematological toxicities were the most commonly reported. There were no treatment related deaths. Toxicity details are reported in Table 2.

**Table 2:** Acute toxicity

Toxicity	G1-G2, n *(%)*	G3-G4, n *(%)*
GI	29 *(38.1)*	5 *(6.6)*
GU	23 *(30.3)*	0 *(0)*
Haematological	14 *(18.4)*	2 *(2.6)*
Skin	9 *(11.8)*	0 *(0)*
Cardiac	3 *(3.9)*	1 *(1.3)*

*GI* Gastrointestinal, *GU* Genitourinary

### Response to treatment and surgical data

Overall, 72 out of 76 (94.7%) patients underwent surgery consisting of low anterior resection (LAR) in 53 (73.6%), abdomino-perineal resection (APR) in 11 (15.3%) and trans-anal local excision (LE) in 8 (11.1%) patients, respectively. Among the remaining four patients, three with lower rectum cancer and complete clinical response at post-CT-RT re-staging refused surgery and one patient showed a systemic progression of disease after CT-RT. Postoperative complications at 30 days after operation were reported in 13 (18%) patients. In particular, 3 (4.2%) patients underwent surgery for small-bowel obstruction, anastomotic leakage and pelvic abscess, respectively. Further details are reported in Table 3. The median time from the end of the CT-RT to surgery was 10.7 weeks (range 6.4–12.4 weeks).

**Table 3:** Postoperative complications

Surgery (n. of patients)	Anastomotic leakage	Bleeding	Pelvic abscess	Small-bowel obstruction	Others	Total, n *(%)*
LAR (53/72)	1	2	3	2	3	11 *(15.3)*
APR (11/72)	0	0	1	0	1	2 *(2.8)*
LE (8/72)	0	0	0	0	0	0 *(0)*
Total, n *(%)*	1 *(1.4)*	2 *(2.8)*	4 *(5.6)*	2 *(2.8)*	4 *(5.6)*	13 *(18.1)*

Tumour and lymph node downstaging was documented in 51 (70.8%) of 72 operated patients, including LE, and in 43 (67%) of 64 radically operated patients, respectively. Overall, 46 (63.9%) patients achieved a major pathologic response (TRG1–2). In particular the tumor pCR (pT0) was documented in 20 (27.8%) out of 72 patients and 14 (22%) out of 64 radically operated patients achieved a pCR (pT0N0). Response to treatment is reported in Table 4. Circumferential resection margin (CRM) was positive in 3 (4%) out of the 72 surgically treated patients. The 3 patients account for 9% of the 34 with MRF involvement.

**Table 4:** Response to treatment: T/N down-staging and TRG

	pT0	pT1	pT2	pT3	pT4	Total, n *(%)*
cT2	2	0	1	0	0	3 *(4.2)*
cT3	17	8	17	19	0	61 *(84.7)*
cT4	1	0	2	4	1	8 *(11.1)*
Total, n *(%)*	20 *(27.8)*	8 *(11.1)*	20 *(27.8)*	23 *(31.9)*	1 *(1.4)*	72 *(100)*
	pN0	pN1	pN2	pNx^a^	Total, n *(%)*	
cN0	7	2	1	2	12 *(16.7)*	
cN1	22	9	1	5	37 *(51.4)*	
cN2	19	2	1	1	23 *(31.9)*	
Total, n *(%)*	48 *(66.7)*	13 *(18.1)*	3 *(4.2)*	8 *(11.1)*	72 *(100)*	
	TRG1	TRG2	TRG3	TRG4	TRG5	Total, n *(%)*
cT2	2	1	0	0	0	3 *(4.2)*
cT3	17	21	21	2	0	61 *(84.7)*
cT4	1	4	3	0	0	8 *(11.1)*
Total, n *(%)*	20 *(27.8)*	26 *(36.1)*	24 *(33.3)*	2 *(2.8)*	0 *(0)*	72 *(100)*

^a^Patient underwent local excision

Adjuvant chemotherapy was planned in patients with pathologic positive lymph nodes by all participating centers. Overall, 16 (22%) out 72 patients received adjuvant chemotherapy with Capecitabine 2000 mg/m2/daily for 14 days, q 3 weeks. Capecitabine was given according to the planned time and dose to all 16 patients.

The potential impact of some patient and tumor characteristics and of the different IMRT-SIB dose levels on TRG1 (pT0) and tumor downstaging were evaluated by both univariate and multivariate analyses. No difference for any of the considered prognostic factors was reported, except for the distance of lower pole of tumor from EAS ≥ 60 mm (OR = 0.12). Also, no difference was reported for the IMRT-SIB dose levels when compared by chi-square test (*p* = 0.47) and evaluated by the multivariate analysis (Table 5).

**Table 5:** Odds ratios (OR) and corresponding 95% confidence intervals (CI) for TRG1 and tumor downstaging according to selected characteristics

	TRG 1		OR (95% CI)^a^	Downstaging		OR (95% CI)^a^
	n	(%)		n	(%)	
Sex						
Man	13	(26.0)	Reference	34	(70.8)	Reference
Woman	7	(26.9)	1.67 (0.60–4.62)	18	(72.0)	0.90 (0.21–3.88)
Age (years)						
< 55	5	(29.4)	Reference	12	(70.6)	Reference
55–64	5	(18.5)	0.36 (0.10–1.27)	14	(56.0)	0.37 (0.07–1.99)
65–74	6	(31.6)	1.01 (0.26–3.85)	16	(84.2)	2.93 (0.40–21.37)
≥ 75	4	(30.8)	0.85 (0.20–3.56)	10	(83.3)	2.75 (0.34–22.48)
Distance EAS (mm)						
< 60	9	(23.7)	Reference	29	(80.6)	Reference
≥ 60	8	(27.6)	0.88 (0.30–2.55)	17	(58.6)	0.12 (0.02–0.69)
Unknown	3	(33.3)	1.026 (0.22–7.12)	6	(75.0)	0.15 (0.01–2.43)
cT						
cT2	2	(40.0)	Reference	3	(100.0)	-
cT3	17	(27.0)	0.29 (0.02–4.14)	42	(67.7)	-
cT4	1	(12.5)	0.29 (0.02–5.68)	7	(87.5)	-
cN						
cN0	4	(33.3)	Reference	8	(66.7)	Reference
cN1	10	(25.6)	0.59 (0.16–2.23)	25	(67.6)	0.39 (0.06–2.60)
cN2	6	(24.0)	0.77 (0.18–3.34)	19	(79.2)	1.14 (0.17–7.82)
MRF involvment						
No	6	(17.7)	Reference	31	(79.5)	Reference
Yes	14	(33.3)	0.34 (0.11–1.01)	21	(61.8)	0.29 (0.07–1.18)
Single dose intensity						
2.1	3	(18.8)	Reference	12	(80.0)	Reference
2.16	9	(37.5)	1.45 (0.36–5.84)	15	(62.5)	0.16 (0.02–1.47)
≥ 2.2	8	(22.2)	1.45 (0.39–5.38)	25	(73.5)	1.14 (0.14–9.25)

^a^Adjusted for all variables reported in the table

## Discussion

Given the reported relationship between RT-delivered dose and tumor response, RT intensification represents an attractive possibility to improve the clinical outcome in LARC. The recent results from the phase III INTERACT trial demonstrating an equivalent efficacy, with better compliance and tolerance of RT intensification using 3D–CRT concomitant boost with Capecitabine-based CT compared with CT intensification with Capecitabine plus Oxaliplatin and standard RT, prompted our interest to investigate treatment optimization programs in RT intensification with IMRT for LARC [13, 19–23].

In our experience on 76 LARC patients, the treatment appeared to be feasible and effective. IMRT-SIB with a dose range of 52.5–57.5Gy (median 54Gy) and Capecitabine with 1650 mg/m^2^/daily resulted in a low rate of toxicity; grade ≥ 3 gastrointestinal (GI) and overall grade ≥ 3 toxicity was reported in only 6.6% and 10.5% patients, respectively. These data appear closely comparable with the other available IMRT-SIB intensification and Capecitabine phase II studies or retrospective series [24–29]. These studies documented grade ≥ 3 acute toxicity rates of 4–25% with a dose range of 47.5–57.5Gy (median 55Gy); therefore our results confirm the optimal tolerance of this treatment intensification modality. Interestingly, a higher incidence with 25% of major toxicity was reported by Arbea et al. [29] when Oxaliplatin was added to Capecitabine, requiring RT dose attenuation (from 2.5Gy to 2.4Gy/fraction), thus suggesting the potential limitations in feasibility of IMRT-SIB with multidrug chemotherapy. The 6.6% rate of grade ≥ 3 GI acute toxicity is significantly lower than the 12.6%–20% rate of the preoperative CT-RT with two-drug CT arm (5FU or Capecitabine plus Oxaliplatin and 3D–CRT of 50.4Gy), whereas is similar to the 3.2%–15% rate of the preoperative standard CT-RT arm (5FU or Capecitabine alone with 3D–CRT of 50.4Gy), which were reported in the more recent randomised trials [3–7]. This favourable toxicity rate is also lower than the 24% rate reported by Myerson et al. [11] and the 15% rate more recently reported by Valentini et al. [13] with RT intensification using 3D–CRT concomitant boost technique and Capecitabine. This remarkable difference could be mainly attributed to a significantly decreased incidence of diarrhea as a result of improved small bowel sparing with IMRT-SIB technique as reported by Samuelian et al. [30] in their retrospective study comparing IMRT to the more conventional 3D–CRT.

The good tolerance to IMRT-SIB allowed a treatment compliance as high as 97.4% for the RT and 84.2% for the CT component, which is well comparable to 93.6% and 91% rates reported with 3D–CRT concomitant boost technique and Capecitabine, respectively (13). These results are consistent with the other published studies with CT-RT intensification using IMRT-SIB, which confirm the feasibility of this treatment approach (Table 6). Overall, in our experience, the compliance of IMRT-SIB and Capecitabine was closely comparable also to the standard 3D–CRT and 5FU or Capecitabine of the control arm (92–100% for RT and 79–97% for CT) reported in the more recent randomized trials [3–7]. In addition, IMRT dose intensification did not adversely affect the compliance to adjuvant chemotherapy, which was 100% for the 16 patients treated after surgery.

**Table 6:** Short-term results of phase II and of retrospective available studies

Author *(* ^*a*^ *Study)*	N. of patients	Stage	Radiotherapy	Compliance RT-CT	pCR	Tox ≥ 3	Post-op morbidity
De Ridder et al. [24] *(Phase II)*	24/13	T3-T4 MRF+	46Gy/23fr/2 *SIB* 55.2Gy/23fr/2.4Gyfr	100% -noCT	14%	4.2%	8%
Li et al. [25] *(Phase II)*	63	T3-T4 N+	41.8Gy/22fr/1.9 *SIB* 50.6Gy/22fr/2.3Gyfr	100% –100%	31%	14%	7%
Hernando et al. [26] *(Retrospective)*	74	T2-T4 N+	45Gy/25fr/1.8 *SIB* 57.5Gy/25fr/2.3Gyfr	99% – 99%	31%	17.6%	7%
Zhu et al. [27] *(Phase II)*	78	T3-T4 N0/N+	50Gy/25fr/2 *SIB* 55Gy/25fr/2.2Gyfr	100% – 62%	24%	14%	17%
Wang et al. [28] *(Retrospective)*	260	T3-T4- N0/N+	41.8Gy/22fr/1.9 *SIB* 50.6Gy/22fr/2.3Gyfr	99% – 99%	18.5%	6%	23%
^b^Arbea et al. [29] *(Phase II)*	100	T3-T4 N+	47.5Gy/19fr/2.5 *and* 47.5Gy/20fr/2.4Gyfr	97% – 80%	13%	25%	7%
Our experience	76	T3-T4 N0/N+	45 Gy/25fr/1.8 *SIB* 52.5–57.Gy/25fr 2.1–2.3Gyfr	97.4% – 84%	22%	10.5%	18%

^a^All Studies included concurrent Capecitabine ^b^Concurrent Capecitabine-Oxaliplatin

Data related to treatment response were encouraging. Tumour and lymph node downstaging was reported in 70.8% and in 67% of patients, respectively, with a pCR (pT0N0) rate of 22%. In addition, the overall tumor pathological complete response (pT0) resulted of 27.8% of cases when all operated patients were considered, including the patients selected for LE (pT0Nx). These results are consistent with pCR rates of 11.5%–19% of the randomised trials, including the INTERACT trial with a pCR and pT0 rates of 23% and 29.8%, respectively (3–7,13). Despite the limitations due to the different RT doses delivered (from 52.5Gy to 57.5Gy, with 2.1Gy–2.3Gy/fraction), our pCR rate of 22% is similar to that reported by other IMRT-SIB intensification series (with doses ranging from 47.5 to 57.5Gy) with a pCR rate of 13–31% [24–29]. Among the studies with a higher rate of pCR, Li et al. [25] reported a 31% pCR rate with concomitant Capecitabine and an IMRT-SIB dose of 50.6 Gy/23 fractions with a single SIB fraction of 2.3 Gy and Hernando-Requejo et al. [26] reported similar results (pCR rate of 31%) with Capecitabine and IMRT-SIB dose of 57.5Gy/25 fraction with a single SIB fraction of 2.3 Gy, indicating a possible correlation between dose fraction and response rate, although these data need to be carefully evaluated because of the limited number of treated patients in these series (Table 6). It should be noted that 90% of patients with MRF involvement at diagnosis had negative CRM after CT-RT.

At multivariated analysis, the only parameter which impacted on TRG1 (pT0) and on tumor downstaging rates was the distance of tumor from EAS, wich is a well-known negative prognostic factor, whereas no impact was reported for the other parameters considered in the analysis, including the different dose levels of RT. However, the small number of patients evaluated in each subset considered may have affected this conclusion. Nevetheless, tolerance and response rate in terms of T and N downstaging and TRG appear to indicate the dose range of 52.5–57.5Gy to be effective with IMRT-SIB for LARC. As for postoperative morbidity, we documented a complication rate of 18%. This data is consistent with the 7–23% rate of the other IMRT-SIB studies (Table 6), and appears well comparable with the 25–36% rate reported with 3D–CRT concomitant boost studies [11, 13]. Interestingly, 8 of 72 (11%) patients underwent LE without significant complications, which suggests the feasibility of organ preservation surgery also after this intensified RT program associated with Capecitabine.

We are aware that our study has several limitations. These include mainly its retrospective nature, the limited number of evaluated patients and the different SIB doses employed due to the different IMRT modalities available at each participating centre. Although patient distribution for the several parameters evaluated at multivariate analysis was quite balanced, the limited sample size might represent a possible explanation for the lack of any significant indication, including the RT dose level. In addition, although all the five participating centres had collaborated on the previous INTERACT rectal cancer trial and shared the same CTVs delineation criteria, the lack of a centralised review of each case could have impacted on the validity of these results. There was also no central review in Mandard TRG scoring. However, even with these limitations, this is to our knowledge the first pooled analysis of a multicentric experience of preoperative IMRT-SIB and full-dose Capecitabine in LARC.

## Conclusion

In conclusion, in our pooled analysis preoperative IMRT-SIB with dose range of 52.5–57.5 Gy (median 54Gy) in 25 fractions and Capecitabine appeared feasible, safe and effective in terms of downstaging and pCR. These short-term results may contribute to the growing evidence of RT intensification in combination with CT as a promising option in the management of LARC. Long-term toxicity and the impact on disease control and patient survival will be evaluated with a longer follow-up time.

## References

[1] Valentini V, Glimelius B, Haustermans K (2014). EURECCA consensus conference highlights about rectal cancer clinical management: the radiation oncologist's expert review. Radiother Oncol.

[2] Maas M, Nelemans PJ, Valentini V (2010). Long-term outcome in patients with a pathological complete response after chemoradiation for rectal cancer: a pooled analysis of individual patient data. Lancet Oncol..

[3] O’Connell MJ, Colangelo LH (2014). Capecitabine and oxaliplatin in the preoperative multimodality treatment of rectal cancer: surgical end points from National Surgical Adjuvant Breast and bowel project trial R-04. J Clin Oncol.

[4] Rodel C, Liersch T, Becker H (2012). Preoperative chemoradiotherapy and postoperative chemotherapy with fluorouracil and oxaliplatin versus fluorouracil alone in locally advanced rectal cancer: initial results of the German CAO/ARO/AIO-04 randomised phase 3 trial. Lancet Oncol.

[5] Gerard JP, Azria D, Gourgou-Bourgade S (2010). Comparison of two neoadjuvant chemoradiotherapy regimens for locally advanced rectal cancer: results of the phase III trial ACCORD 12/0405-Prodige 2. J Clin Oncol.

[6] Aschele C, Cionini L, Lonardi S (2011). Primary tumor response to preoperative chemoradiation with or without oxaliplatin in locally advanced rectal cancer: pathologic results of the STAR-01 randomized phase III trial. J Clin Oncol.

[7] Schmoll H-J, Haustermans K, Price TJ (2013). Preoperative chemoradiotherapy and postoperative chemotherapy with capecitabine and oxaliplatin versus capecitabine alone in locally advanced rectal cancer: first results of the PETACC-6 randomized phase III trial. J Clin Oncol.

[8] Burbach JP, den Harder AM, Intven M, van Vulpen M, Verkooijen HM, Reerink O (2014). Impact of radiotherapy boost on pathological complete response in patients with locally advanced rectal cancer: a systematic review and meta-analysis. Radiother Oncol.

[9] Appelt AL, Pløen J, Vogelius IR, Bentzen SM, Jakobsen A (2013). Radiation dose-response model for locally advanced rectal cancer after preoperative chemoradiation therapy. Int J Radiat Oncol Biol Phys.

[10] Suwinski R, Taylor JM, Withers HR (1998). Rapid growth of microscopic rectal cancer as a determinant of response to preoperative radiation therapy. Int J Radiat Oncol Biol Phys.

[11] Myerson RJ, Valentini V, Birnbaum EH (2001). A phase I/II trial of three-dimensionally planned concurrent boost radiotherapy and protracted venous infusion of 5-FU chemotherapy for locally advanced rectal carcinoma. Int J Radiat Oncol Biol Phys.

[12] Krishnam S, Janjan NA, Skibber JM (2006). Phase II study of capecitabine (Xeloda) and concomitant boost radiotherapy in patients with locally advanced rectal cancer. Int J Radiat Oncol Biol Phys.

[13] Valentini V, De Paoli A, Barba MC, et al. Capecitabine-based preoperative chemo-radiotherapy in rectal cancer intensified by RT dose or Oxaliplatin: the INTERACT trial. Radiother Oncol. 2014; 111, Supplement 1, S:181.

[14] Teoh S, Muirhead R (2016). Rectal radiotherapy- intensity-modulated radiotherapy delivery, delineation and doses. Clin Oncol (R Coll Radiol).

[15] Engels B, Platteaux N, Van den Begin R (2014). Preoperative intensity-modulated and image-guided radiotherapy with a simultaneous integrated boost in locally advanced rectal cancer: report on late toxicity and outcome. Radiother Oncol.

[16] Cilla S, Caravatta L, Picardi V (2012). Volumetric modulated arc therapy with simultaneous integrated boost for locally advanced rectal cancer. Clin Oncol (R Coll Radiol).

[17] MERCURY (2007). Study group: extramural depth of tumor invasion at thin-section MR in patients with rectal cancer: results of the Mercury study. Radiology.

[18] Mandard AM, Dalibard F, Mandard JC (1994). Pathological assessment of tumour regression after preoperative chemoradiotherapy of esophageal carcinoma. Clinicopathological correlations Cancer.

[19] Arbea L, Ramos LI, Martínez-Monge R, Moreno M, Aristu J (2010). Intensity-modulated radiation therapy (IMRT) vs. 3D conformal radiotherapy (3DCRT) in locally advanced rectal cancer (LARC): dosimetric comparison and clinical implications. Radiat Oncol.

[20] Richetti A, Fogliata A, Clivio A (2010). Neo-adjuvant chemo-radiation of rectal cancer with volumetric modulated arc therapy: summary of technical and dosimetric features and early clinical experience. Radiat Oncol.

[21] Mok H, Crane CH, Palmer MB (2011). Intensity modulated radiation therapy (IMRT): differences in target volumes and improvement in clinically relevant doses to small bowel in rectal carcinoma. Radiat Oncol.

[22] Liu M, Liu B, Wang H (2015). Dosimetric comparative study of 3 different postoperative radiotherapy techniques (3D-CRT, IMRT, and RapidArc) for II-III stage rectal cancer. Medicine (Baltimore).

[23] Duthoy W, De Gersem W, Vergote K (2004). Clinical implementation of intensity-modulated arc therapy (IMAT) for rectal cancer. Int J Radiat Oncol Biol Phys.

[24] De Ridder M, Tournel K, Van Nieuwenhove Y (2008). Phase II study of preoperative helical tomotherapy for rectal cancer. Int J Radiat Oncol Biol Phys.

[25] Li JL, Ji JF, Cai Y (2012). Preoperative concomitant boost intensity-modulated radiotherapy with oral capecitabine in locally advanced mid-low rectal cancer: a phase II trial. Radiother Oncol.

[26] Hernando-Requejo O, Lopez M, Cubillo A (2014). Complete pathological responses in locally advanced rectal cancer after preoperative IMRT and integrated-boost chemoradiation. Strahlenther Onkol.

[27] Zhu J, Liu F, Gu W (2014). Concomitant boost IMRT-based neoadjuvant chemoradiotherapy for clinical stage II/III rectal adenocarcinoma: results of a phase II study. Radiat Oncol.

[28] Wang L, Li ZY, Li ZW, et al. Efficacy and safety of neoadjuvant intensity-modulated radiotherapy with concurrent capecitabine for locally advanced rectal cancer. Dis *Colon rectum* 2015; 58(2):186-92. 10.1097/DCR.0000000000000294 25585076

[29] Arbea L, Martínez-Monge R, Díaz-González JA (2012). Four-week neoadjuvant intensity-modulated radiation therapy with concurrent capecitabine and oxaliplatin in locally advanced rectal cancer patients: a validation phase II trial. Int J Radiat Oncol Biol Phys.

[30] Samuelian JM, Callister MD, Ashman JB, Young-Fadok TM, Borad MJ, Gunderson LL (2012). Reduced acute bowel toxicity in patients treated with intensity-modulated radiotherapy for rectal cancer. Int J Radiat Oncol Biol Phys.

